# Management of glycemic variation in diabetic patients receiving parenteral nutrition by continuous subcutaneous insulin infusion (CSII) therapy

**DOI:** 10.1038/s41598-018-24275-5

**Published:** 2018-04-12

**Authors:** Feng-fei Li, Wen-li Zhang, Bing-li Liu, Dan-feng Zhang, Wei Chen, Li Yuan, Mao-yuan Chen, Xiao-fang Zhai, Jin-dan Wu, Xiao-fei Su, Lei Ye, Hong-yong Cao, Jian-hua Ma

**Affiliations:** 1 Department of Endocrinology, Nanjing First Hospital, Nanjing Medical University, Nanjing, China; 2 Department of Surgery, Nanjing First Hospital, Nanjing Medical University, Nanjing, China; 3 National Heart Research Institute Singapore, National Heart Centre, Singapore, Singapore

## Abstract

To compare the continuous subcutaneous insulin infusion (CSII) or insulin glargine based multiple injections (MDI) therapy on glycemic variations in diabetic patients receiving PN outside of intensive care settings. This was a single-center, randomized, open-label trial. Patients with type 2 diabetes (T2D) who were receiving parenteral nutrition (PN) were recruited. After baseline data were collected, recruited patients were then randomized 1:1 to a CSII group or a MDI group. All patients were subjected to a 4-day retrospective Continuous Glucose Monitoring (CGM). The primary endpoint was the differences of the 24-hrs mean amplitude of glycemic excursion (MAGE) in patients receiving the PN therapy between the two groups. A total of 102 patients with T2D receiving PN were recruited. Patients in the CSII group had a significantly decreased mean glucose level (MBG), the standard deviation of MG (SDBG), MAGE, and the coefficient of variation (CV%) compared to those in MDI group (all P < 0.01). Furthermore, we found that the patients who received a bolus insulin dose required maintaining euglycemic control was gradually decreased during the PN period in both groups at the endpoint. The administration of insulin via CSII led to a significant decrease in glycemic variations in patients receiving PN.

## Introduction

Surgical patients are at increased risk of malnutrition, in particular, hyperglycemia^1^. Nearly half of the patients who were receiving parenteral nutrition had hyperglycemia (PN) outside of the intensive care settings^2,3^. Hyperglycemia is positively associated with increased hospital complications and mortality for patients receiving PN^4^. Patients receiving PN with glycaemia above 180 mg/dL had increased death rates ranging from 2- to >10-fold and increased complications than those with mean blood glucose levels less than 140 mg/dL^4–6^. Therefore, clinical practice guidelines and consensus statements recommend a blood glucose levels had points of 140–180 mg/dL patients who are receiving PN^7,8^.

Prandial and neutral protamine Hagedorn (NPH) insulin administration was the usually preferring protocol in management of blood glucose in patients receiving PN. To providing better control of blood glucose control, researchers prefer to administer only part of insulin in the PN bag, and the other part of insulin administered subcutaneously^9^.

Insulin dose is significantly increased during administration of PN for management of glycemic control in patients with type 2 diabetes (T2D)^10^. The reason may partly be that patients needing PN support are associated with a raised level of stress, which leads to hyperglycemia in some patients^11–13^. In addition to this, the increased levels of cortisol and catecholamines induced by surgical procedures leading to significantly increased insulin resistance, also^14^. Hyperglycemic, especially an acute glucose spike which triggers oxidative stress, and an over-production of peroxynitrite and nitrotyrosine^15–17^, is an independent risk factor for cardiovascular disease^18^. This is significant factor to consider, and that makes it necessary to take steps to avoid blood glucose fluctuations during the management of PN. Continuous subcutaneous insulin infusion (CSII) is a safe therapy to smooth blood glycemic execution during the management of hyperglycemia either in newly diagnosed or in longstanding T2D patients^19^.

Continuous Glucose Monitoring (CGM) monitoring of glucose concentrations every 5 min could be a useful method for discovering the 24-hrs glucose profiles in patients receiving PN. Using CGM data, clinicians could efficiently evaluate the glycemic control which might be an important factor to consider in decision-making^20^.

However, it is unknown the efficacy of CSII therapy on glycemic variation in patients receiving PN outside of intensive units. In the current study, we performed 4-day CGM to assess the effect of CSII on blood glucose fluctuations in surgery patients receiving PN.

## Results

### Baseline characteristics

Between May 2017 and Aug 2017, a total of 102 patients were consecutively recruited for the study, and randomized to CSII therapy group (n = 50) and MDI group (n = 52). There were no significant demographic differences within groups at baseline, with the exception of body weight and BMI in MDI group were significantly lower than that in CSII group. Also, there were no differences in HbA_1c_, mean fasting blood glucose, and mean fasting plasma C-Peptide levels between groups (Table 1).

**Table 1:** Demographic characteristics in subjects between groups at baseline.

	CSII Group (n = 50)	MDI Group (n = 52)	*P* value
Sex (male)	68.0%	59.6%	0.38
Age (years)	66.8 ± 9.1	65.3 ± 9.3	0.42
Body weight (Kg)	64.0 ± 8.9	60.1 ± 5.4	0.01
Height (cm)	165.7 ± 5.7	163.9 ± 5.6	0.12
BMI (kg/m^2^)	23.34 ± 3.15	22.34 ± 1.51	0.04
Course (years)	5.2 ± 3.0	5.7 ± 2.5	0.33
HbA_1c_ (%)	7.7 ± 1.4	8.2 ± 1.2	0.07
FBG (mmol/L)	9.6 ± 3.74	8.67 ± 1.83	0.11
F C-peptide (ng/mL)	1.7 ± 1.7	1.9 ± 0.9	0.59
Operative site (S/I/P/L)	40%/40%/4%/16%	48.1%/32.7%/5.8%/13.5%	0.80

Data were presented as means ± SD. BMI: Body mass index, Course: Course of diabetes, FBG: Fasting blood Glucose level, F C-peptide: Fasting C-Peptide concentration, S: Stomach, I: Intestine, P: Pancreas, L: Liver.

All patients receiving PN therapy in both groups had had gastrointestinal surgery. There were no differences in surgery sites between the two groups (CSII group: stomach 40%/intestine 40%/pancreas 4%/liver 16%, MDI group: stomach 48.1%/intestine 32.7%/pancreas 5.8%/liver 13.5%, P > 0.05).

Before operation, patients in the CSII group reached glycemic control in fewer days than those in the control group (2.23 ± 1.82 vs. 4.32 ± 1.17 days, P < 0.05). In addition, patients in the CSII group needed similar daily insulin dose to maintain glycemic control compared to those in the MDI group (0.43 ± 0.22 vs. 0.46 ± 0.25 IU/Kg, P > 0.05).

### 24-hrs Glycemic variation profiles

**Figure 1:**
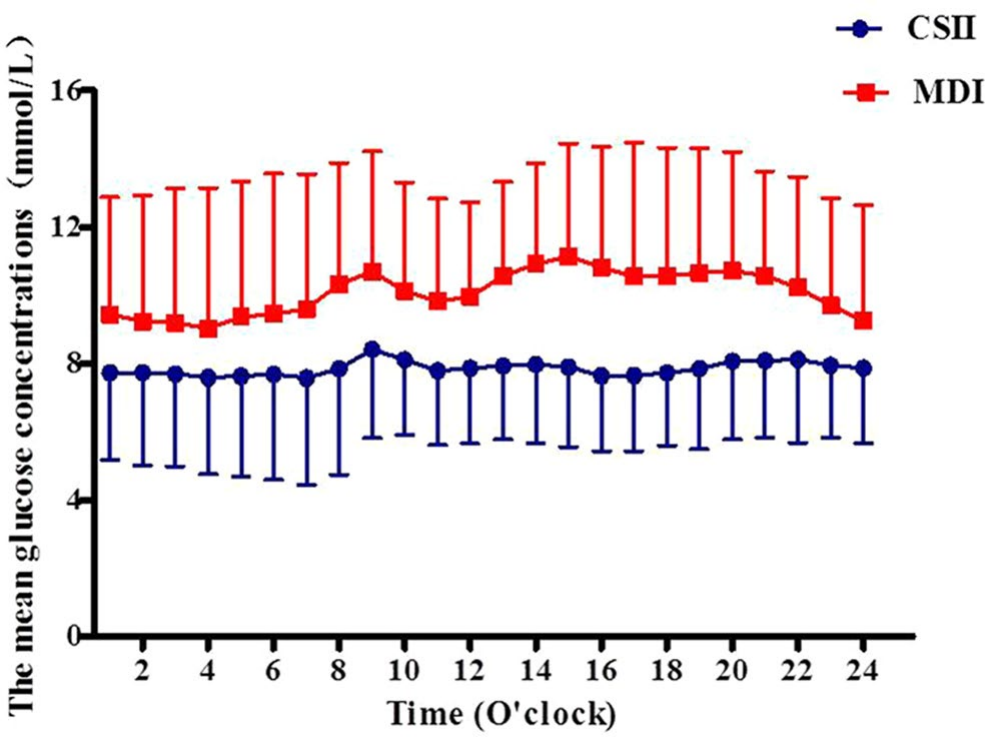
The hourly glucose concentrations calculated from CGM in subjects between groups at the endpoint.

Patients receiving the CSII therapy had significantly decreased in the 24-hrs mean amplitude of glycemic excursions (MAGE), the standard deviation of MG (SDBG), SD, the coefficient of variation (CV%), the 24-hrs mean blood glucose (MBG), and the incremental area under the curve (AUC) > 10 mmol/L compared to those in MDI group, respectively (all P < 0.01). We did not observe any differences in the incremental AUC  < 3.9 mmol/L between groups (P > 0.05) (Table 2). We also observed that subjects in the CSII group exhibited significantly lower hourly mean glucose levels compared to those in the MDI group, respectively (all P < 0.05) (Table 3 and Fig. 1).

**Table 2:** Glycemic profiles between groups in study subjects at the endpoint.

	CSII Group (n = 50)	MDI Group (n = 52)	*P* value
24 hrs-AUC > 10	3.3(0.0,282.0)	2024.5(574.4,3253.5)	0.00
24 hrs-AUC < 3.9	0.0(0.0,0.0)	0.0(0.0,9.9)	0.84
24 hrs-SD	1.4 ± 1.0	2.5 ± 1.0	0.00
24 hrs-MAGE	3.7 ± 2.8	6.2 ± 3.0	0.00
24 hrs-MBG	7.0 ± 1.9	10.1 ± 2.4	0.00
24 hrs- CV (%)	17.6 ± 11.4	26.7 ± 11.9	0.00
14 hrs-AUC > 10	0.0(0.0,24.2)	223.6(53.5,396.3)	0.00
14 hrs-AUC < 3.9	0.0(0.0,0.0)	0.0(0.0,0.0)	0.96
14 hrs-SD	1.1 ± 0.9	2.1 ± 1.0	0.00
14 hrs- CV (%)	13.8 ± 9.9	21.7 ± 11.1	0.00

Data were presented as means ± SD or IQR. 24-hrs: Glycemic profiles of 24-hrs, AUC > 10: the incremental area under curve of glucose concentrations above 10 mmol/L (mmol/L per day), AUC < 3.9: the incremental area under curve of glucose concentrations less than 3.9 mmol/L (mmol/L per day), SD: standard deviation (mmol/L), MAGE: mean amplitude of glycemic excursions (mmol/L), MBG: mean glucose concentration (mmol/L), CV: coefficient of variation (%), 14-hrs: Glycemic profiles of 14-hrs (During PN period).

**Table 3:** Hourly mean glucose concentration in subjects between groups at the endpoint.

Time (O’clock)	CSII Group (n = 50)	MDI Group (n = 52)	*P* value
0000–0100 (mmol/L)	7.7 ± 2.6	9.4 ± 3.4	0.01
0100–0200 (mmol/L)	7.7 ± 2.7	9.2 ± 3.7	0.02
0200–0300 (mmol/L)	7.7 ± 2.7	9.2 ± 3.9	0.03
0300–0400 (mmol/L)	7.6 ± 2.8	9.0 ± 4.1	0.04
0400–0500 (mmol/L)	7.6 ± 2.9	9.4 ± 4.0	0.01
0500–0600 (mmol/L)	7.7 ± 3.1	9.5 ± 4.1	0.02
0600–0700 (mmol/L)	7.6 ± 3.1	9.6 ± 4.0	0.01
0700–0800 (mmol/L)	7.8 ± 3.1	10.3 ± 3.6	0.00
0800–0900 (mmol/L)	8.4 ± 2.6	10.7 ± 3.5	0.00
0900–1000 (mmol/L)	8.1 ± 2.2	10.1 ± 3.2	0.00
1000–1100 (mmol/L)	7.8 ± 2.2	9.8 ± 3.0	0.00
1100–1200 (mmol/L)	7.9 ± 2.2	10.0 ± 2.8	0.00
1200–1300 (mmol/L)	7.9 ± 2.1	10.6 ± 2.8	0.00
1300–1400 (mmol/L)	8.0 ± 2.3	10.9 ± 2.9	0.00
1400–1500 (mmol/L)	7.9 ± 2.3	11.2 ± 3.3	0.00
1500–1600 (mmol/L)	7.6 ± 2.2	10.8 ± 3.5	0.00
1600–1700 (mmol/L)	7.7 ± 2.2	10.6 ± 3.9	0.00
1700–1800 (mmol/L)	7.7 ± 2.1	10.6 ± 3.8	0.00
1800–1900 (mmol/L)	7.6 ± 2.4	10.7 ± 3.7	0.00
1900–2000 (mmol/L)	8.1 ± 2.3	10.7 ± 3.5	0.00
2000–2100 (mmol/L)	8.1 ± 2.2	10.6 ± 3.1	0.00
2100–2200 (mmol/L)	8.1 ± 2.4	10.2 ± 3.2	0.00
2200–2300 (mmol/L)	7.9 ± 2.1	9.7 ± 3.2	0.00
2300–2400 (mmol/L)	7.9 ± 2.2	9.3 ± 3.4	0.01

Data were presented as means ± SD. Course: Course of diabetes, S: Stomach, I: Intestine, P: Pancreas, L: Liver.

### 14-hrs Glycemic variation profiles (During PN time)

To observe the impaction of dextrose containing in Fatemulsion, Aminoacids (17) And Glucose (11) Injection, we also analyzed glycemic profiles of patients during the PN period. Our CGM data showed that patients in CSII therapy had significant improvement in 14-hrs MBG, MAGE, SDBG, SD, GV(%), and the incremental AUC >10 mmol/L compared to those in the MDI group, respectively (all P < 0.01). We did not observe any patients who experienced hypoglycemia in either group (P > 0.05) (Table 3). In addition, patients in the CSII group had significantly lower hourly mean glucose levels when compared to those in the MDI group (all P < 0.05) (Table 3 and Fig. 1).

### Glucose and insulin profile

Patients receiving CSII therapy needed significantly less insulin to maintain glycemic control when compared to those in the MDI group (P < 0.01) at the endpoint. Consistently, the basal and bolus insulin doses were also decreased in the CSII therapy group (P < 0.01). However, in this trial, recruited patients in the CSII group had lower body weight compared to those in the MDI group. We further analyzed the insulin dose per Kg between groups. Our data showed that patients in the CSII group required significantly lower basal and bolus insulin doses (IU per Kg) when compared to the control group to maintain glycemic control at the endpoint, P < 0.01. In addition to this, we also observed that patients who received bolus insulin doses which were required to maintain euglycemic control had their doses gradually decreased before the end point of PN. This was observed in both groups (1000–1600 *vs*. 1600–2000, 1600–2000 *vs*. 2000–2400, P < 0.01, respectively) (Table 4).

**Table 4:** Insulin dose required by patients to maintain glycemic control in subjects between groups at the endpoint.

	CSII Group (n = 50)	MDI Group (n = 52)	*P* value
TOD (IU)	25.3 ± 5.0	32.3 ± 2.9	0.00
1000–2400 (IU)	21.6 ± 4.1	26.1 ± 2.4	0.00
0000–1000 (IU)	3.8 ± 1.2	6.2 ± 0.7	0.00
0000–1600 (IU)	12.2 ± 2.0	16.2 ± 1.5	0.00
1600–2000 (IU)	5.5 ± 1.4	7.4 ± 1.0	0.00
2000–2400 (IU)	3.8 ± 1.1	2.5 ± 0.3	0.00
TOD/Kg (IU/Kg)	0.4 ± 0.1	0.5 ± 0.1	0.00
1000–2400 (IU/Kg)	0.3 ± 0.1	0.4 ± 0.1	0.00
0000–1000 (IU/Kg)	0.1 ± 0.0	0.1 ± 0.0	0.00
1000–1600 (IU/Kg)	0.2 ± 0.0	0.3 ± 0.0	0.00
1600–2000 (IU/Kg)	0.1 ± 0.0	0.1 ± 0.0	0.00
2000–2400 (IU/Kg)	0.1 ± 0.0	0.0 ± 0.0	0.00

TOD: Total daily dose.

### Safety and tolerance

No episodes of hypoglycemia requiring medical assistance were reported in either group. All subjects were well served by PN during the study, and no infection event was reported in the two groups.

## Discussion

The management of hyperglycemia during PN was primarily focused on protocol-guided insulin dosing therapy in non-critical care settings^21^ or a low-calorie PN regimen^22^. There are few studies that have addressed glycemic variation management during PN in diabetic patients.

We conducted a prospective study on patients receiving PN and demonstrated that the CSII therapy could significantly reduce insulin doses and provided further improvement of glycemic fluctuations. Previous studies to assess glycemic control were done mainly by utilizing intermittent fingerpicks. Thus, the 24-hrs blood glycemic excursions are undoubtedly missed by these point-to-point glimpses of blood glucose. CGM provides a unique opportunity to examine the 24-hrs glucose excursions in diabetic patients receiving PN. In the present pilot study, we expected to see better improvement of glycemic variations in the CSII group when compared to the insulin glargine based multiple injections regimen group because the CSII therapy could provide more flexible ways for insulin infusion being compared to insulin administered via injection. Our CGM data showed that patients receiving CSII therapy had significant improvement in MAGE, SD and CV% when compared to those treated with an insulin glargine based multiple injections regimen. GV could be presented by a group of indices originated from the CGM in diabetic patients^23–25^, such as SD and MAGE, and there is a high degree of correlation between SD and MAGE^26,27^. In this study, the lower SD and MAGE in patients with CSII therapy might contribute to the beneficial effects seen in microvascular and macrovascular complications. It has been observed that acute glucose fluctuations other than chronic hyperglycemia have been shown to play an important role in oxidative stress^16^ and nitrosative stress^15–17^. Interestingly, the improvement of glycemic variations could lead to a significant decreasing of oxidative and nitrosative stress^28^. However, in this study, we did not monitor the level of oxidative and nitrosative stresses. We addressed these as limitations in this study. In addition, our data showed that patients receiving the CSII therapy reached glycemic goals significantly earlier than in the MDI groups, and this was in agreement with our previous observations which indicated that newly diagnosed or longstanding T2D patients needed a significantly shorter time to achieve euglycemic control by receiving the CSII therapy when compared to those with MDI therapy^19^. Our study demonstrated that insulin administration was not the provider of beneficial effects seen with morbidity and mortality^29^. On the contrary, insulin administration played a detrimental role in mortality in patients receiving PN^30^. In this study, insulin administered via the CSII therapy which significantly decreased insulin doses when compared to those of glargine based multiple injections groups. Thus, we could infer that the decreased insulin doses administration might contribute to the benefits seen with morbidity and mortality. Future studies are needed to identify the insulin doses which were administered and which had adverse outcomes in patients receiving PN in non-intensive care settings.

It’s well established that an elevation in blood glucose was a risk factor for infection^31–33^. However, we did not observe any infections in this study in either group. This may be a shortcoming of our study, and we are now recognizing this as another limitation of our study.

There is a controversy in the history of diabetes as to whether or not taking a course which could result in additional risk of complications in patients receiving PN should be followed^4,5,34^. A previous study reported that patients with a history of diabetes had higher levels of blood glucose levels than those in another group^4^. We did not observe a higher incidence of hyperglycemia in patients with T2D when patients were compared to others. This might be the results of a modest sample and the limitations of study period.

We also provided a pattern of insulin doses required by patients to achieve the euglycemic control during PN. In this study, our observations were that patients receiving PN required more different pattern of insulin doses to maintain glycemic control, especially during the last 4-hrs period of PN therapy. The reason may partially be the absorption of insulin by PN bags. The percentage lost is very low 90–95%^35^. Another observation of importance was that we observed that patients in both groups needed significantly gradually decreased bolus insulin doses to maintain euglycemic control at the endpoint. Future study is needed to identify the discharge of insulin attached to the bags in different infusion time-point during PN. Insulin therapy carries an increase in risks of hypoglycemia and weight gain^36–38^. However, in this study, subjects in both groups had no events of hypoglycemia or glucose levels less than 3.9 mmol/L monitored by CGM.

The study of the patient population was limited to the Nanjing area in China, and we recognize the results might be different in other geographical regions or populations.

In conclusion, the administration of insulin via CSII led to a significant decrease in glycemic variations, and insulin doses required by patients receiving PN to maintain euglycemic control when compared to the MDI therapy in patients with T2D who had had gastrointestinal surgery.

## Methods

**Figure 2:**
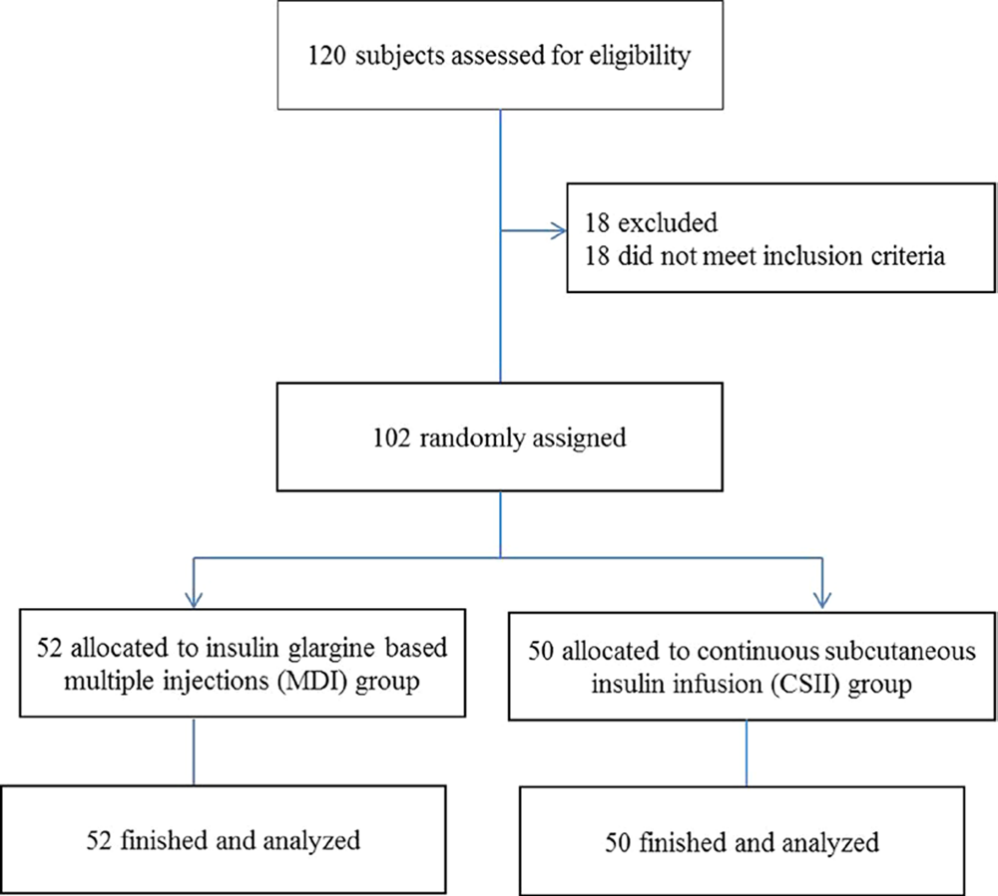
Study flow chart.

This was a single-center, randomized, open-label trial. The study protocol and patient consent forms were approved by the Institutional Ethical Committee of Nanjing First Hospital, Nanjing Medical University. All procedures followed were in accordance with the ethical standards of Nanjing First Hospital, and with the Helsinki Declaration of 1964 as revised in 2013. Informed consent was obtained from all patients for being included in the study. The study flow chart was described as Fig. 2.

Between May 2017 and Aug 2017, a total of 102 patients with T2D receiving PN were recruited in Nanjing First Hospital, Nanjing Medical University, China. The inclusion criteria were (1) patients aged between 18 and 80 years; (2), confirmed T2D with history or HbA1c ≥6.5%; (3), body mass index (BMI): 21 to 35 kg/m^2^. Patients were excluded if they had a blood glucose concentration >200 mg/dL, chronic kidney disease, or were unwilling to participate.

After baseline data were collected, recruited subjects were then randomized in equal numbers to a CSII group or an insulin glargine based MDI without any oral antidiabetic agent for 3–5 days for achieving glycemic control before surgical treatments. When euglycemic control was achieved, operation was performed and treatments were unchanged during the study period in the two groups. From day 1 after surgery, patients were received Fatemulsion, Aminoacids (17) glucose (11) Injection infusions (Kabiveil PI, Sino-Swed Pharmaceutical Corp. Ltd, Wuxi, China) 1440 ml daily, which contains 96 g dextrose. Insulin was added into Kabiveil PI at the ratio of 1:4 to dextrose (24 units insulin were added into the PN) as previously prescribed^39^. All PN infusions were started daily at 10:30 and run at a rate of 110 mL/h. Followed by Kabiveil PI infusion, a 5% Glucose Injection Solution (500 mL) was supplied to ensure that the amount of infused dextrose to 150 g/d per day^40^. The amount of macronutrient composition was calculated by a PN pharmacist based on each patient’s clinical requirements.

Capillary Blood glucose level was tested every 4-hrs by finger prick to titrate insulin dose with a goal of keeping values 4.6–7.8 mmol/dL. Insulin was administered at 1000 in both groups. The insulin Aspart (Novo Nordisk, Bagsværd, Denmark) dose was 0.05 IU/kg/h in CSII group, which was given in two injection modes: 0.01 IU/kg/h was given after PN therapy, the remaining insulin dose was given as a bolus during PN period. Investigators titrated insulin doses every 4-hrs on an individual-patient basis at the titration algorithm. The total daily insulin dose was also 0.5 IU/kg in MDI group, which was given in two injection modes: 1/5 of total daily insulin dose was administered before bedtime as basal dose (Lantus SoloStar; Sanofi-Aventis, Paris, France), the remaining insulin dose was given as a bolus dose (insulin Aspart) based on blood glucose concentrations monitored at 4-hrs interval during PN period. Investigators titrated insulin doses on an individual-patient basis at the titration algorithm (if the blood glucose level was less than 4.4 mmol/L, the basal insulin dose was reduced 2 units; if the blood glucose level was within 4.4 to 6.1 mmol/L, the basal insulin dose was unchanged; if the blood glucose level was within 6.2 to 7.8, 7.9 to 10.0, and >10.0 mmol/L, the basal insulin dose was increased subsequently by 2, 4, and 6 units, respectively), as we described previously^19^.

All patients were subjected to a 4-day retrospective CGM (Medtronic Incorporated, Northridge, USA) from the day euglycemic control achieved, which was performed as previously described^19,41^. After the CGM data was collected, the 24-hrs MG, the SDBG, and the incremental AUC of glucose above 10.0 mmol/L or less than 3.9 mmol/L, and the hourly MBG, the SDBG, and the CV% were calculated by software provided by Medtronic Incorporated, USA. The MAGE was calculated manually for each patient by measuring the arithmetic mean of the ascending and descending excursions between consecutive peaks and nadirs for the same 24-hrs period, and only absolute excursion values >1 SD were considered, as described previously^19,41,42^.

To observe the efficacy of CSII or MDI therapy on glycemic variations, the 14-hrs MBG, SDBG, CV(%), MAGE, the incremental AUC of glucose above 10.0 mmol/L or less than 3.9 mmol/L, and the hourly MG during PN period were also recorded and analyzed.

Serum samples were obtained before Kabiveil PI administration for glucose, HbA_1c_ and C-peptide concentration determination. HbA_1c_ was measured by a DiaSTAT HbA_1c_ analyzer (Bio-Rad, Hercules, CA). C-peptide and glucose concentrations were measured centrally at the central laboratory in Nanjing First Hospital, Nanjing Medical University.

The primary endpoint was the MAGE in patients receiving PN therapy. Secondary endpoints were the 24-hrs MG, SDBG, incremental AUC of blood glucose above 10.0 mmol/L or less than 3.9 mmol/L, hourly MG, and insulin doses required by patients to maintain glycemic control.

### Statistical Analysis

Statistical analysis was performed using the SPSS software (version 17.0; SPSS, Inc., Chicago, IL). Shapiro-Wilk test was used to assess the distribution of data. Normally distributed and continuous variables are presented as mean (standard deviation, SD). The mixed ANOVA model (2 × 2) test was used to compare the differences within a group. A two way ANOVA was used in the comparisons between groups. Bonferroni correction was followed. P values were two-tailed with a significant level of 5%.

## References

[1] Guidelines for the use of parenteral and enteral nutrition in adult and pediatric patients. *JPEN J Parenter Enteral Nutr* **26**, 1SA–138SA (2002). 11841046

[2] Kim H (2003). Association of hyperglycemia and markers of hepatic dysfunction with dextrose infusion rates in Korean patients receiving total parenteral nutrition. Am J Health Syst Pharm.

[3] Pleva M, Mirtallo JM, Steinberg SM (2009). Hyperglycemic events in non-intensive care unit patients receiving parenteral nutrition. Nutr Clin Pract.

[4] Cheung NW, Napier B, Zaccaria C, Fletcher JP (2005). Hyperglycemia is associated with adverse outcomes in patients receiving total parenteral nutrition. Diabetes care.

[5] Lin LY, Lin HC, Lee PC, Ma WY, Lin HD (2007). Hyperglycemia correlates with outcomes in patients receiving total parenteral nutrition. Am J Med Sci.

[6] Olveira G (2013). Parenteral nutrition-associated hyperglycemia in non-critically ill inpatients increases the risk of in-hospital mortality (multicenter study). Diabetes care.

[7] Moghissi ES (2009). American Association of Clinical Endocrinologists and American Diabetes Association consensus statement on inpatient glycemic control. Diabetes care.

[8] Umpierrez GE (2012). Management of hyperglycemia in hospitalized patients in non-critical care setting: an endocrine society clinical practice guideline. The Journal of clinical endocrinology and metabolism.

[9] Leahy JL (2006). Insulin management of diabetic patients on general medical and surgical floors. Endocr Pract.

[10] Park RH (1992). Management of diabetic patients requiring nutritional support. Nutrition.

[11] Dungan KM, Braithwaite SS, Preiser JC (2009). Stress hyperglycaemia. Lancet.

[12] McCowen KC, Bistrian BR (2004). Hyperglycemia and nutrition support: theory and practice. Nutr Clin Pract.

[13] McCowen KC, Malhotra A, Bistrian BR (2001). Stress-induced hyperglycemia. Crit Care Clin.

[14] Ljungqvist O, Jonathan E (2012). Rhoads lecture 2011: Insulin resistance and enhanced recovery after surgery. JPEN J Parenter Enteral Nutr.

[15] Ceriello A (2008). Oscillating glucose is more deleterious to endothelial function and oxidative stress than mean glucose in normal and type 2 diabetic patients. Diabetes.

[16] Monnier L (2006). Activation of oxidative stress by acute glucose fluctuations compared with sustained chronic hyperglycemia in patients with type 2 diabetes. Jama.

[17] Hu Y, Liu W, Huang R, Zhang X (2010). Postchallenge plasma glucose excursions, carotid intima-media thickness, and risk factors for atherosclerosis in Chinese population with type 2 diabetes. Atherosclerosis.

[18] Nakagami T (2004). Hyperglycaemia and mortality from all causes and from cardiovascular disease in five populations of Asian origin. Diabetologia.

[19] Li FF (2016). Blood Glucose Fluctuations in Type 2 Diabetes Patients Treated with Multiple Daily Injections. Journal of diabetes research.

[20] Rodbard D (2009). Interpretation of continuous glucose monitoring data: glycemic variability and quality of glycemic control. Diabetes technology & therapeutics.

[21] Umpierrez GE (2007). Randomized study of basal-bolus insulin therapy in the inpatient management of patients with type 2 diabetes (RABBIT 2 trial). Diabetes care.

[22] Ahrens CL (2005). Effect of low-calorie parenteral nutrition on the incidence and severity of hyperglycemia in surgical patients: a randomized, controlled trial. Crit Care Med.

[23] Fabris C (2014). Glucose variability indices in type 1 diabetes: parsimonious set of indices revealed by sparse principal component analysis. Diabetes technology & therapeutics.

[24] Fabris C (2015). Parsimonious Description of Glucose Variability in Type 2 Diabetes by Sparse Principal Component Analysis. J Diabetes Sci Technol.

[25] Rodbard D (2009). New and improved methods to characterize glycemic variability using continuous glucose monitoring. Diabetes technology & therapeutics.

[26] Rodbard D (2009). Improved quality of glycemic control and reduced glycemic variability with use of continuous glucose monitoring. Diabetes technology & therapeutics.

[27] Rodbard D (2013). Increased glycemic variability at the onset and during progression of type 2 diabetes-commentary. Diabetes technology & therapeutics.

[28] Ceriello A (2002). Role of hyperglycemia in nitrotyrosine postprandial generation. Diabetes care.

[29] Van den Berghe G (2003). Outcome benefit of intensive insulin therapy in the critically ill: Insulin dose versus glycemic control. Crit Care Med.

[30] Finney SJ, Zekveld C, Elia A, Evans TW (2003). Glucose control and mortality in critically ill patients. Jama.

[31] Pomposelli JJ (1998). Early postoperative glucose control predicts nosocomial infection rate in diabetic patients. JPEN J Parenter Enteral Nutr.

[32] Golden SH, Peart-Vigilance C, Kao WH, Brancati FL (1999). Perioperative glycemic control and the risk of infectious complications in a cohort of adults with diabetes. Diabetes care.

[33] Zerr KJ (1997). Glucose control lowers the risk of wound infection in diabetics after open heart operations. Ann Thorac Surg.

[34] Sarkisian S, Fenton TR, Shaheen AA, Raman M (2010). Parenteral nutrition-associated hyperglycemia in noncritically ill inpatients is associated with higher mortality. Can J Gastroenterol.

[35] Marcuard SP, Dunham B, Hobbs A, Caro JF (1990). Availability of insulin from total parenteral nutrition solutions. JPEN J Parenter Enteral Nutr.

[36] Nathan DM (2009). Medical management of hyperglycemia in type 2 diabetes: a consensus algorithm for the initiation and adjustment of therapy: a consensus statement of the American Diabetes Association and the European Association for the Study of Diabetes. Diabetes care.

[37] Charbonnel B, Cariou B (2011). Pharmacological management of type 2 diabetes: the potential of incretin-based therapies. Diabetes, obesity & metabolism.

[38] Barnett AH (2010). Key considerations around the risks and consequences of hypoglycaemia in people with type 2 diabetes. Int J Clin Pract.

[39] Valero MA (2001). Evaluation of nonglucose carbohydrates in parenteral nutrition for diabetic patients. Eur J Clin Nutr.

[40] McClave SA (2009). Guidelines for the Provision and Assessment of Nutrition Support Therapy in the Adult Critically Ill Patient: Society of Critical Care Medicine (SCCM) and American Society for Parenteral and Enteral Nutrition (A.S.P.E.N.). JPEN J Parenter Enteral Nutr.

[41] Li FF (2015). Influence of Acarbose on Plasma Glucose Fluctuations in Insulin-Treated Patients with Type 2 Diabetes: A Pilot Study. Int J Endocrinol.

[42] Li FF (2017). Continuous Glucose Monitoring in Newly Diagnosed Type 2 Diabetes Patients Reveals a Potential Risk of Hypoglycemia in Older Men. Journal of diabetes research.

